# Development of high slip-resistant footwear outsole using rubber surface filled with activated carbon/sodium chloride

**DOI:** 10.1038/s41598-021-04102-0

**Published:** 2022-01-07

**Authors:** Toshiaki Nishi, Takeshi Yamaguchi, Kazuo Hokkirigawa

**Affiliations:** 1 Institute of Sport Science, ASICS Corporation, 6-2-1, Takatsukadai, Nishi-ku, Kobe, Hyogo 651-2271 Japan; 2 Department of Finemechanics, Graduate School of Engineering, Tohoku University, 6-6-01 Aramaki Aza-Aoba, Aoba-ku, Sendai, 980-8579 Japan; 3 Graduate School of Biomedical Engineering, Tohoku University, 6-6-01 Aramaki Aza-Aoba, Aoba-ku, Sendai, 980-8579 Japan

**Keywords:** Risk factors, Materials science, Physics

## Abstract

High slip-resistant footwear outsoles can reduce the risk of slip and fall on wet and icy surfaces. Falls on wet and icy surfaces can cause serious life-threatening injuries, especially for older adults. Here we show that footwear outsoles using the rubbers filled with activated carbon or sodium chloride produce higher friction force and reduce the slip rate in walking. We have identified that small depressions were formed on outsole materials filled with activated carbon or sodium chloride during friction between the rubber and surface leading to some air ingress into the interface. While there are air bubbles between the rubber and surface, real contacts are surrounded by water with negative pressure (Laplace pressure). It is considered that the negative pressure promotes real contact formation, which causes high friction. We consider that the outsole materials filled with activated carbon or sodium chloride can reduce the risk of slip-and-fall accidents.

## Introduction

Slip-and-fall accidents can be a risk factor for serious injuries in daily life^1^. This risk can be reduced by improving slip-resistant outsoles on slippery floors such as wet smooth floors^2^. Thus, there has been many attempts to improve the slip resistance of outer soles by structure designing^3–5^ and material designing^6^. On wet floors, water intervention causes low real contact area, which results in low friction^7^. In the friction of soft matter such as outsole rubber and water between two substrates can be eliminated based on the surface free energy called the dewetting effect^8,9^. In addition, it would be meaningful to make a wetting state non-uniform because non-uniform wetting promotes real contact formation, which enlarges friction coefficient *μ*^10,11^. Here, non-uniform wetting is defined by a heterogeneous interface between the rubber (hydrophobic) and hydrogel patch (hydrophilic) on floor^10^ and the heterogeneous interface between rubber and floor, which contains air bubbles (hydrophobic) in water (hydrophilic)^11^. When the diameter of pores on hydrophobic material is less than 10–100 μm, water cannot penetrate into the pores^12^; thus, non-uniform wetting can be achieved with the existing air pockets (small pores) on the rubber surface. Here, the influence of roughness of rubber surface on friction behaviour has been reported^13,14^, but, to enclose air in the pores, the size of pores is needed to control in smaller scale (10–100 μm) in comparison with this previous study. Considering the wear of rubber surface containing air pockets in a practical use of outsole, it is better to add small gaps that can turn into air pockets by the atmosphere. Here, activated carbon (AC) and sodium chloride (SC) particles can be suitable candidates as filler materials that create air pockets on the rubber surfaces. AC is one of the carbon-based porous materials and some pores are expected to form on the rubber outsole surface with the addition of AC; moreover, since the water-soluble SC on the rubber surface can be removed by exposing it to water, air pockets form on the rubber surface. Additionally, AC and SC are chemically and thermally stable and do not affect chemical reactions in the cross-linking process; therefore, rubber with AC or SC can be useful for outsole materials.

Different types of filler materials such as carbon black and silica have been added to rubber to improve the stiffness, tensile strength, tear strength, and wear resistance^15–17^. Even the specific surface area of carbon black is not smaller than that of AC, AC is chemically similar to carbon black^18^. Here, AC can be produced from biomass materials, such as wood, crop residues, sawdust, coconut shell, animal waste, aquatic plants, algae, tannery solid waste, dead leaf, and bamboo^19–24^. Thus, to replace carbon black to AC in a view point of environment protection, the physical properties of rubber with AC has been eagerly investigated^24,25^, but the friction behaviour of rubber with AC has not been reported, On the other hand, SC has not reinforcing effect and the friction behaviour of rubber with SC has not also been reported; thus, this study aims to develop the slip resistance of outsole rubber materials filled with AC or SC and to clarify real contact formation and friction behaviour between these rubbers and floors. Therefore, vulcanised isoprene rubber (IR) with/without AC or SC were prepared at different contents controlling the elastic modulus *E*, and hemispheres and outsoles were prepared using these materials. The friction test using rubber hemispheres and outsoles, and slip-resistance test using outsoles in stepping motion was conducted.

## Results and discussion

### Friction behaviours of hemisphere specimens

#### Effects of AC or SC addition on friction coefficient under wet conditions

**Figure 1:**
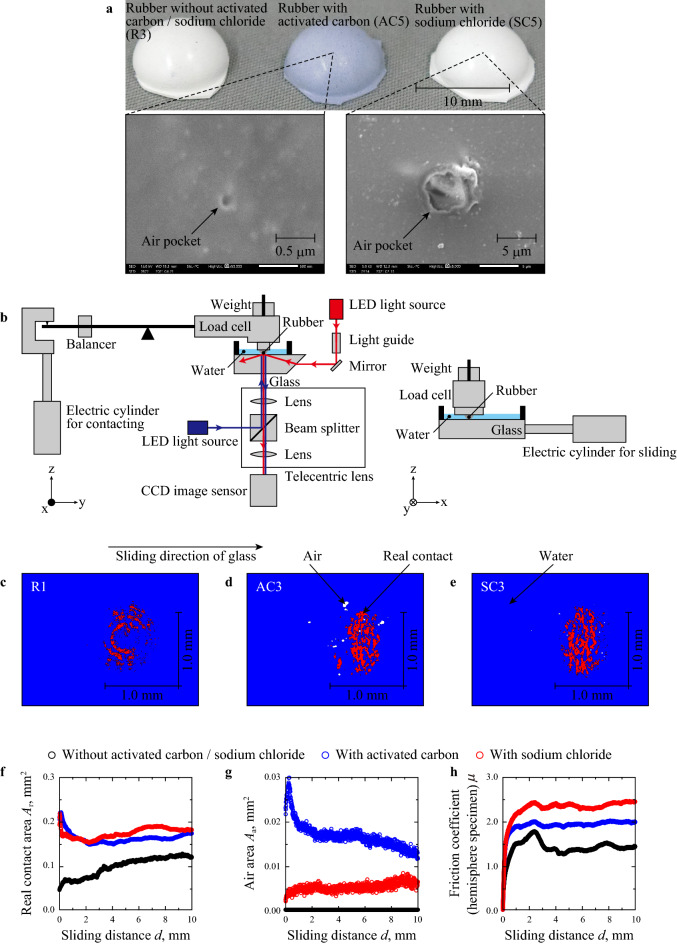
Overview of hemisphere specimens and obtained results in friction test using hemisphere specimens. (**a**) Images of hemisphere specimens (R3, AC5, and SC5). (**b**) Schematic of the experimental system for friction test. (**c**–**e**) Distribution of real contact, air bubble and water at *d* = 5.00 mm for R1, AC3 and SC3. (**f**–**h)**, Changes in the real contact area (*A*_r_), air area (*A*_a_) and friction coefficient (*μ*) for R1, AC3 and SC3.

**Figure 2:**
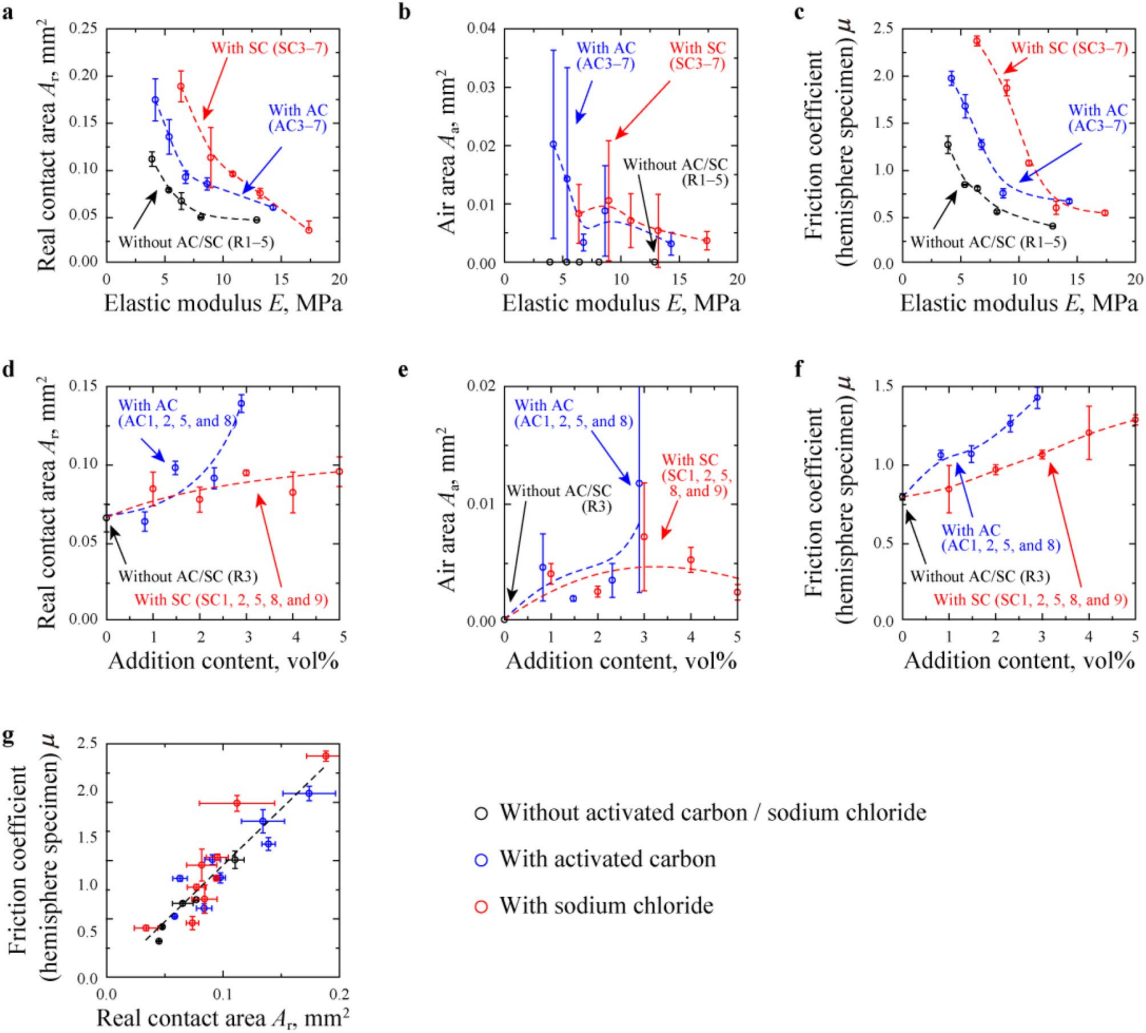
Measured parameters in friction test using hemisphere specimens. (**a**–**c**) Influence of elastic modulus (*E*) on the real contact area (*A*_r_), air area (*A*_a_) and friction coefficient (*μ*) for R1–5, AC3–7 and SC3–7. **(d**–**f**) *A*_r_, *A*_a_ and *μ* versus the content of AC and SC for R3, AC1, AC2, AC5, AC8, SC1, SC2, SC5, SC8 and SC9. (**g**) Relationship between *μ* and *A*_r_.

The outsole rubber materials filled with AC or SC (Fig. 1a) were prepared, as listed in Table 1, and the contact condition and friction force of these samples were measured during friction on water-covered glass (Fig. 1b). Here, localization of air pockets (pinholes) for rubber with AC or SC were not observed in Fig. 1a. Considering the median size of AC was about 50 μm, most of AC surface was covered with rubber matrix, but partially protrudes to the surface. And, there were air pockets corresponding to the size of SC particles (the median size of SC was 11.1 μm). Figure 1c–e show the distribution of real contacts (red area), air-covered area (white area) and water-covered area (blue area) for R1, AC3 and SC3 at *d* = 5.00 mm, respectively. Here, the results at these conditions in Fig. 1c–e are shown as representative results. For all rubbers including the other rubber in Fig. 1c–e, many real contacts were formed within an elliptic shape whose minor axis was parallel to the sliding direction. These results resulted in the macroscopic strain in each hemisphere specimen as reported by Sahli et al.^26,27^. The air bubbles were formed only at rubber filled with AC and SC. It is considered that the air contained in each air bubble leaked from the air pockets on the rubber surface (Fig. 1a). Even after the friction test using hemisphere specimens was continuously conducted 5 times within five minutes, air bubbles were always observed for rubber filled with AC and SC. This result explains that the air in the air pockets was not completely replaced with water and continuously supplied air to the interface between the two substrates. Focusing on the outer edge of real contacts, it is confirmed that the outer edge did not contact with air bubbles, in other words, each real contact was surrounded by water. Similar trend has been also reported in the previous study^11^. Figure 1f–h reveal the changes in real contact area *A*_r_, air area (air-covered area) *A*_a_ and friction coefficient *μ* during the friction test using hemisphere specimens for R1, AC3 and SC3. It can be observed that the *A*_r_ value of R1 has clearly increased in the range of sliding distance *d* = 1.00–10.00 mm; however, the variation of *μ* for R1 was relatively unstable at *d* = 0.00–5.00 mm, while it showed a steady trend at *d* = 5.00–10.00 mm. As seen from Fig. 1g, the *A*_a_ values are 0.00 mm^2^ at *d* = 0.00–10.00 mm. The *A*_r_ values of AC3 and SC3 rubber materials decreased at *d* = 0.00–1.00 mm and then slightly increased at *d* = 1.00–10.00 mm, while *μ* remained constant values during the friction test at *d* = 2.00–10.00 mm. Interestingly, the *A*_a_ values of AC3 and SC3 are not zero during the friction test, which indicates that the air bubbles were trapped within the apparent contact area during a sliding process as shown in Fig. 1d,e. Because, the value of $\gamma_{{{\text{AirWater}}}}$ (corresponding to $\gamma_{{{\text{Water}}}}$ = 72.8 mJ/m^2^) is larger than $\gamma_{{{\text{AirRubber}}}}$ (corresponding to $\gamma_{{{\text{Rubber}}}}$ = 24.2–26.0 mJ/m^2^) and $\gamma_{{{\text{AirGlass}}}}$ (corresponding to $\gamma_{{{\text{Glass}}}}$ = 51.5 mJ/m^2^), the total free energy can be minimised when the air bubbles contact with the surfaces of rubber and glass, which would enable the attachment of air bubbles within the apparent contact area. Figure 2a–c indicate mean values and standard distribution of *A*_r_, *A*_a_ and *μ* respectively, for R1–5 (rubbers without AC or SC), AC3–7 (rubbers with AC) and SC3–7 (rubbers with SC) plotted against the elastic modulus *E*. It was observed that *A*_r_ values of all rubbers have decreased with the increase in *E*. While *A*_r_ at 6.4 < *E* < 6.8 MPa increased for + 39 and + 188% by adding AC and SC, respectively, the increasing rates of *A*_r_ at 12.9 < *E* < 14.3 MPa were + 30 and + 64% by adding AC and SC, respectively. In addition, the *A*_a_ values of R1 fluctuated around 0 in the range of 0–20 MPa since it does not contain any air bubbles. However, the *A*_a_ values of AC and SC are > 0 due to having air bubbles and decreased with the increase in *E*. The negative correlations between *A*_r_ and *E* can be explained on the basis of Hertz contact theory wherein low *E* enlarges apparent contact area *A*_0_^28^. The negative correlations between *A*_a_ and *E* for rubbers filled with AC and SC can also be explained on the basis of Hertz contact theory, because the increase in *A*_0_ would enlarge potential area where air bubbles were formed. Regarding the dependency of *A*_r_ on *E*, Fig. 2c illustrates that there are negative correlations between *μ* and *E*, and that *μ* has increased by adding AC and SC, especially at *E* < 10 MPa. Figure 2d–f reveal the values of *A*_r_, *A*_a_ and *μ* for R3 (rubbers without AC or SC), AC1, AC2, AC5, AC8 (rubbers filled with AC), SC1, SC2, SC5, SC8 and SC9 (rubbers filled with SC) plotted against AC or SC content. Figure 2e illustrates that air bubbles were formed by adding AC and SC, while the *A*_a_ values showed up-down trends with AC and SC contents; however, as observed in Fig. 2d,f, *A*_r_ and *μ* values have increased with the increase in AC and SC contents. Figure 2g reveals the proportional relationship between *μ* and *A*_r_ for all conditions. This result demonstrates that the real contact formation was promoted by adding AC and SC, which leads to increase in *μ* due to improvements in adhesion^29,30^; thus, the real contact formation was promoted by adding AC and SC, which increased *μ*.

**Table 1:** Content of AC or SC and physical properties among prepared rubbers.

	AC content (vol%)	SC content (vol%)	Elastic modulus (MPa)	Tensile strength (MPa)	Tear strength (N/mm)	Surface free energy *γ* (mJ/m^2^)	Spreading coefficient contacting with glass in water *S* (mJ/m^2^)
R1	0.0	0.0	5.6	14.0	75.9	24.2	− 29.9
R2	0.0	0.0	8.7	14.4	77.3	24.7	− 29.9
R3	0.0	0.0	9.9	17.9	78.0	25.9	− 31.4
R4	0.0	0.0	12.5	19.9	85.5	24.9	− 30.0
R5	0.0	0.0	15.4	16.7	72.3	26.0	− 31.5
AC1	0.83	0.0	6.6	20.0	70.9	24.2	− 29.9
AC2	1.5	0.0	6.6	16.3	69.0	24.8	− 29.9
AC3	2.4	0.0	4.2	14.2	36.4	25.7	− 31.4
AC4	2.5	0.0	5.4	14.7	43.1	24.2	− 29.9
AC5	2.3	0.0	6.8	14.6	57.9	26.0	− 31.5
AC6	2.2	0.0	8.6	14.5	68.8	25.7	− 31.4
AC7	2.3	0.0	14.3	15.6	74.8	25.8	− 31.4
AC8	2.9	0.0	6.9	12.4	39.7	25.8	− 31.4
SC1	0.0	1.0	10.9	17.0	75.7	25.3	− 30.0
SC2	0.0	2.0	10.2	18.1	74.5	24.6	− 29.9
SC3	0.0	3.0	6.4	18.3	77.2	24.6	− 29.9
SC4	0.0	3.0	8.9	16.5	71.3	24.5	− 29.9
SC5	0.0	3.0	10.8	16.8	77.0	24.9	− 31.3
SC6	0.0	3.0	13.2	16.5	70.9	24.8	− 29.9
SC7	0.0	3.0	17.4	14.4	75.5	25.1	− 31.4
SC8	0.0	4.0	10.8	16.0	71.7	24.5	− 29.9
SC9	0.0	4.0	11.3	14.0	67.9	24.2	− 29.9

#### The high friction mechanism of rubber with AC or SC

**Figure 3:**
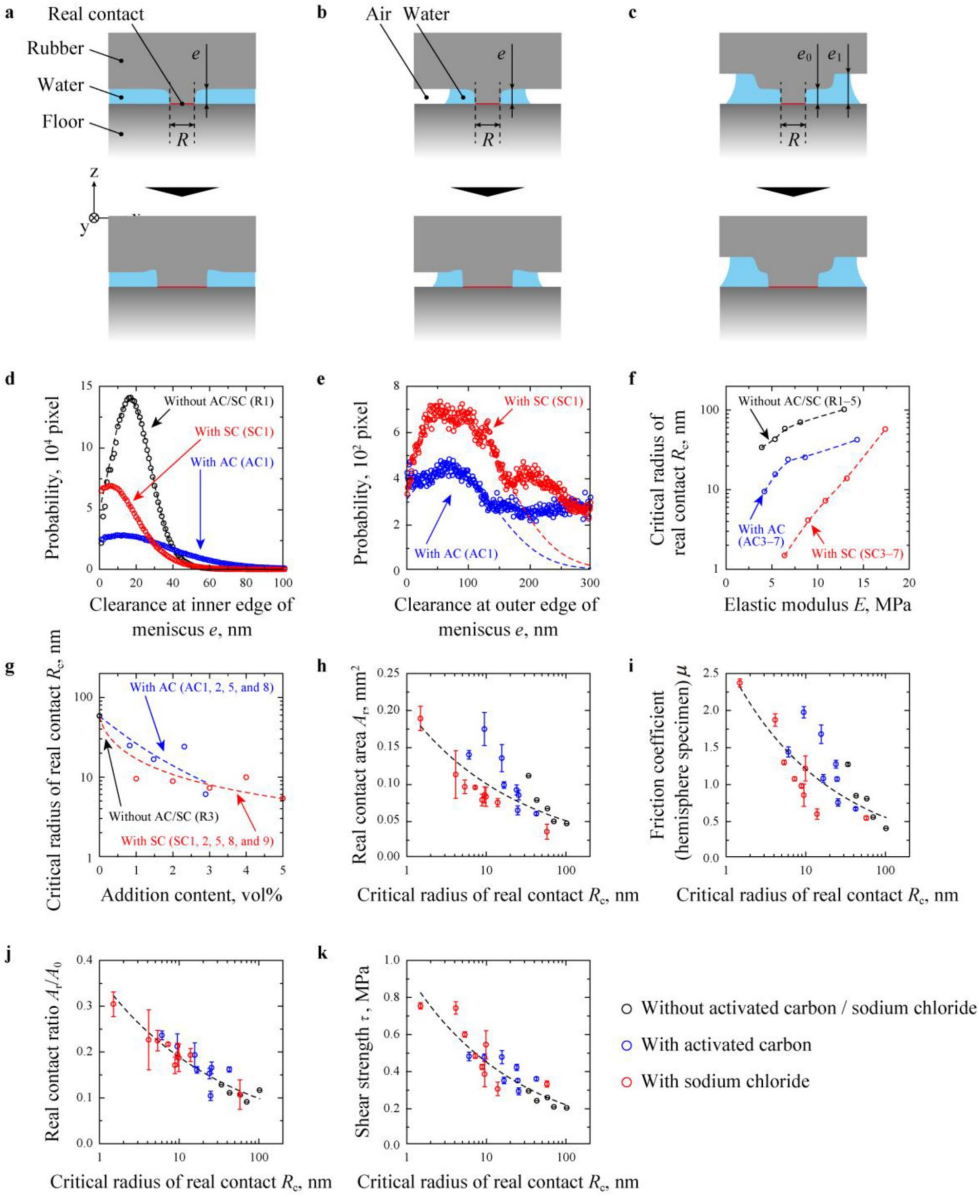
Obtained parameters relating to dewetting effect. (**a**–**c**) Schematic of real contact formation based on the dewetting effect: (**a**) uniform wetting; (**b**) non-uniform wetting but uniform clearance in the water meniscus; (**c**) non-uniform wetting and non-uniform clearance in the water meniscus. (**d**, **e**) Histograms of clearance at the inner (**d**) and outer (**e**) edges of the meniscus for R1, AC3 and SC3. (**f**, **g**) Critical radius of real contact (*R*_c_) plotted against elastic modulus (*E*) for R1–5, AC3–7 and SC3–7 and addition content for R3, AC1, AC2, AC5, AC8, SC1, SC2, SC5, AC8, and SC9. (**h**, **i**) Real contact area (*A*_r_) and friction coefficient (*μ*) versus *R*_c_. (**j**, **k**) Real contact ratio (*A*_r_/*A*_0_) and shear strength (*τ*) versus *R*_c_.

According to Eqs. (1) and (2), real contacts of soft material on a floor as shown in Fig. 3a thermodynamically expand when the spreading coefficient *S* has a negative value in water or the work of adhesion *W* has a positive value in air, and this phenomenon is called a dewetting effect^8,9,31^. *S* and *W* correspond to the parameter of quantifying wettability between rubber and floor under un-lubricated and lubricated conditions, respectively, and these parameters can be calculated from the following equations^12,32^:

1 $$W = \left( {\gamma_{{\text{R}}} + \gamma_{{\text{G}}} } \right) - \gamma_{{{\text{RG}}}}$$

2 $$S = \gamma_{{{\text{RG}}}} - \left( {\gamma_{{{\text{RW}}}} + \gamma_{{{\text{GW}}}} } \right)$$

where the subscripts R, G and W correspond to rubber, glass and water, respectively; $\gamma_{{\text{i}}}$ is the surface free energy of material I and $\gamma_{{{\text{ij}}}}$ is the interfacial free energy between materials i and j. In Kaelble and Uy theory, $\gamma_{{\text{i}}}$ is defined as the sum of dispersion term $\gamma_{{\text{i}}}^{{\text{d}}}$ and polar term $\gamma_{{\text{i}}}^{{\text{p}}}$ ($\gamma_{{\text{i}}} = \gamma_{{\text{i}}}^{{\text{d}}} + \gamma_{{\text{i}}}^{{\text{p}}}$), and $\gamma_{{{\text{ij}}}}$ is obtained via the following equations^12,33^:

3 $$\gamma_{{{\text{ij}}}} = \left( {\sqrt {\gamma_{{\text{i}}}^{{\text{d}}} } - \sqrt {\gamma_{{\text{j}}}^{{\text{d}}} } } \right)^{2} + \left( {\sqrt {\gamma_{{\text{i}}}^{{\text{p}}} } - \sqrt {\gamma_{{\text{j}}}^{{\text{p}}} } } \right)^{2}$$

The dewetting effect is explained on the basis of free energy of the system *G,* which is a convex quadratic function of the real contact size *R* in the following equations^8,9^:

4 $$G \propto {-}\left| S \right|R^{2} + Ee^{2} R$$

where *e* indicates the clearance between two substrates. This equation explains that when *R* is higher than *R*_c_ = *Ee*^2^/ǀ*S*ǀ, the real contact thermodynamically expands due to the negative values of *G* and negatively increasing rate of *G*. It has been theoretically and experimentally reported that real contact formation can be promoted as *R*_c_ decreases^11^. In addition, this theory has been developed to explain for a non-uniform wetting state where there are some air bubbles between two substrates in water, and the real contacts are surrounded by water meniscus as shown in Fig. 3b^11^. It has been reported that *G* and *R*_c_ for such a condition are obtained in the following equations^11^:

5 $$G \propto - \left( {2W + S} \right)R^{2} + Ee^{2} R$$

6 $$R_{{\text{c}}} = { }\frac{{Ee^{2} }}{2W + S}.$$

The pressure in water meniscus is negative at cos*θ*_R_ + cos*θ*_G_ > 0, *Ee*^2^/ǀ*S*ǀ > *Ee*^2^/(2* W* + *S*); thus, if cos*θ*_R_ + cos*θ*_G_ > 0, *R*_c_ decreases due to the air bubbles between two substrates in water and dewetting effect can be promoted. Here, *θ*_i_ is the contact angle of water on material i. Equations (5) and (6) also explain that the dewetting effect is sensitive to the value of *e*. However, as shown in Fig. 3c, *e* is not always constant, depending on the volume of water meniscus; thus, the clearance at the outer edge of the water meniscus (*e*_1_) can be larger than the inner edge of the water meniscus (*e*_0_). Considering that the pressure in water meniscus is determined by *e*_1_, the values for *G* and *R*_c_ for *e*_0_ ≠ *e*_1_ can be determined by the following equations:

7 $$G \propto - \left\{ {2\frac{{e_{0} }}{{e_{1} }}W + \left( {2\frac{{e_{0} }}{{e_{1} }} - 1} \right)S} \right\}R^{2} + e_{0}^{2} ER$$

8 $$R_{c} = \frac{{e_{0}^{2} E}}{{2\frac{{e_{0} }}{{e_{1} }}W + \left( {2\frac{{e_{0} }}{{e_{1} }} - 1} \right)S}}.$$

As confirmed in Fig. 1d,e, the real contacts were surrounded by water meniscus for rubber filled with AC and SC; thus, *R*_c_ for rubber filled with AC and SC can be estimated on the basis of Eq. (8), while *R*_c_ for rubber without AC and SC is defined as *R*_c_ = *E* $e_{0}^{2}$/ǀ*S*ǀ. Here, as the volume of water meniscus increases, the outer edge of the water meniscus reaches to the outer edge of apparent contact and *e*_1_ increases to the depth of lubricant on the glass (more than 1 mm), while the value of *e*_0_ would not drastically change. Thus, the increase of volume of water meniscus induces *e*_0_/*e*_1_ ≈ 0, and Eq. (8) approaches to *R*_c_ = *E* $e_{0}^{2}$/ǀ*S*ǀ. On the other hand, if *e*_0_/*e*_1_ > 1 when the volume of water meniscus is enough small, Eq. (8) explains that *R*_c_ decreases and the dewetting effect increases, but such a situation is also up to the surface geometry. Figure 3d illustrates the histogram of *e* around real contacts for hemisphere specimens of R1, AC1 and SC1, and Fig. 3e indicates the histogram of *e* at the outer edge of water meniscus for hemisphere specimens of AC1 and SC1. The value of *e* on each pixel around real contacts and at the outer edge of the water meniscus was extracted from the measured distribution of *e* at *d* = 5.00–10.00 mm using MATLAB software (R2016b, The MathWorks, Inc.). Figure 3d,e show that each histogram has a single peak. In this study, the peak value for histogram at the inner and outer edge of water meniscus was defined as *e*_0_ and *e*_1_, respectively. Figure 3f,g display the influence of *E* and addition amount of AC or SC on *R*_c_, which was calculated from *S*, *W*, *e*_0_ and *e*_1_. It was confirmed that *R*_c_ increased with increase in *E* and decreased with increase in AC and SC content. Especially at *E* < 10 MPa, *R*_c_ drastically decreased for rubbers filled with SC. In Fig. 3h,i, *A*_r_ and *μ* are plotted against *R*_c_. Overall, *A*_r_ and *μ* decreased with increase in *R*_c_. Considering the fact that *A*_r_ and *μ* can increase with the apparent contact area *A*_0_ determined by *E* in the Hertz contact theory^28^, the real contact ratio *A*_r_/*A*_0_ and the shear strength *τ* (friction force divided by *A*_0_) were calculated and plotted against *R*_c_ in Fig. 3j,k. Figure 3j,k indicate negative correlations in *A*_r_/*A*_0_–*R*_c_ and *τ*–*R*_c_ curves. Defining the approximate lines in Fig. 3h–k as exponential function, the correlation coefficients of *A*_r_/*A*_0_–*R*_c_ and *τ*–*R*_c_ curves were 0.82 and 0.81, respectively, while that of *A*_r_–*R*_c_ and *μ*–*R*_c_ curves were 0.59 and 0.61, respectively. These results suggest that real contact formation in apparent contact based on the dewetting effect was promoted as *R*_c_ got decreased and that *τ* increased by promoting the real contact formation. Therefore, considering that the real contact formation was promoted by adding AC or SC to rubber, especially at *E* < 10 MPa, and that *A*_0_ decreased with *E*, it is reasonable to add AC or SC to soft rubber to achieve high friction.

### Friction behaviours of outer soles

**Figure 4:**
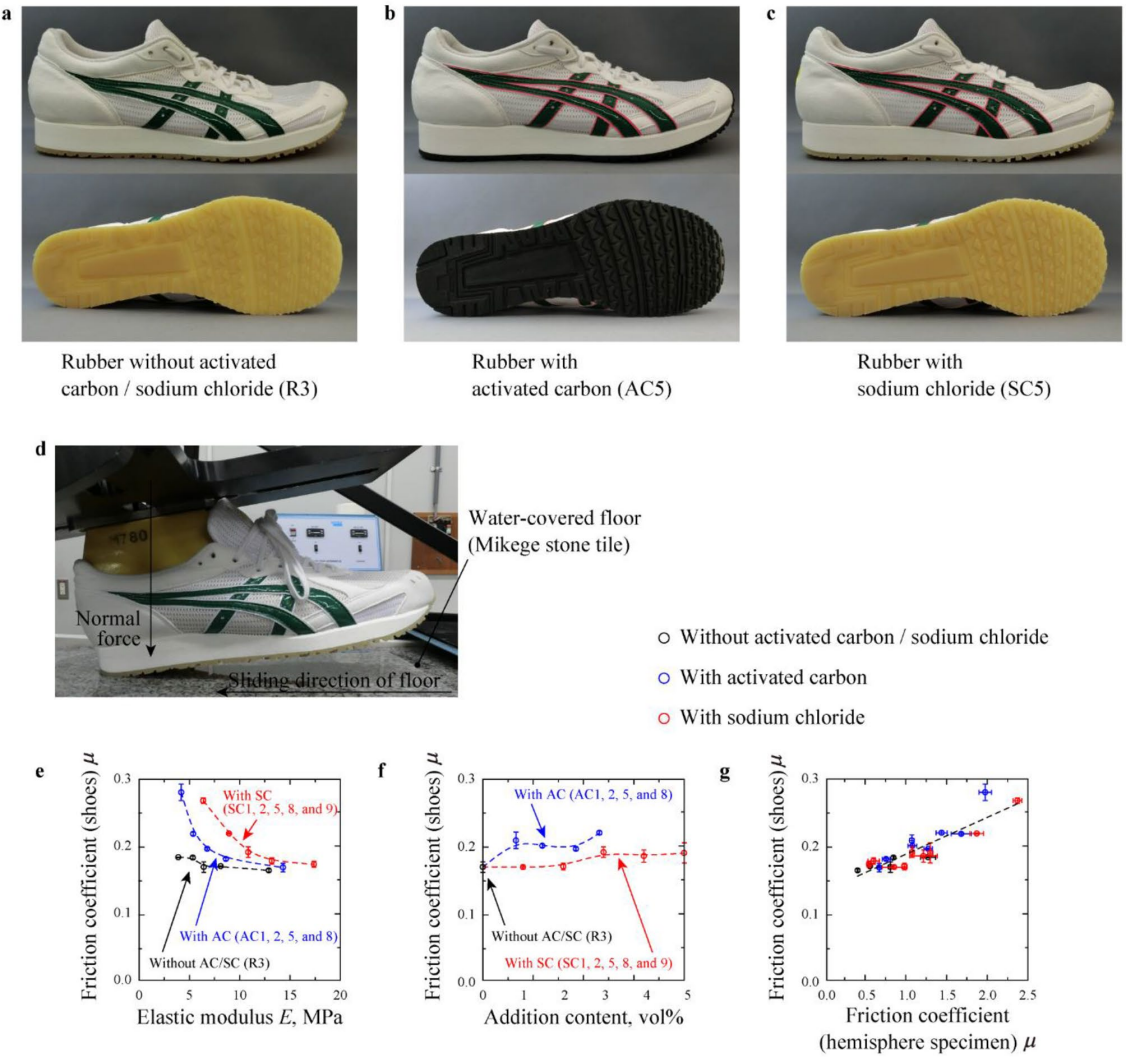
Overview and measured parameters in friction test using shoes. (**a**–**c**) Images of shoes with outer soles of R3, AC5 and SC5. (**d**) Schematic of the experimental system for friction test using shoes with a slip meter. (**e**, **f**) Influences of elastic modulus (E) and AC and SC content on the friction coefficient (*μ*) in friction tests using shoes. **g,** Relationship between *μ* using shoes and hemisphere specimens for all rubbers.

The mounting of footwear outsole rubber materials with/without AC or SC is shown in Fig. 4a–c, and their slip resistance on water-covered smooth floor was measured (Fig. 4d). Figure 4e,f reveal the variation of *μ* values measured using footwear with the dependence of *E* and content of AC and SC. Compared to the results in the friction test using hemisphere specimens, the *μ* values decreased with increase in *E*, increased with the increase in AC or SC content. For the R1, AC3 and SC3 (rubbers made from the softest master batch: MB1), the *μ* increased by + 52.8% and + 46.1% with the addition of AC and SC, respectively, while for R5, AC7, and SC7 (rubbers made from the hardest master batch: MB5), the *μ* increased by + 2.7% and + 5.6% with the addition of AC and SC, respectively. This result explains the softer the higher friction effect of AC and SC addition. Figure 4g indicates a positive correlation between *μ* for footwear outsole materials and hemisphere specimens, but this correlation was not proportional and the value range of *μ* for footwear was minute in comparison with those obtained from hemisphere specimens. These results demonstrate that AC and SC addition enlarged *μ* for footwear, especially at *E* < 10 MPa; however, the absolute values of *μ* for footwear were small because of differences in the contact pressure, the sliding velocity, and the macroscopic geometry in comparison with the friction test using hemisphere specimen.

### Slip rate of shoes in stepping trial

**Figure 5:**
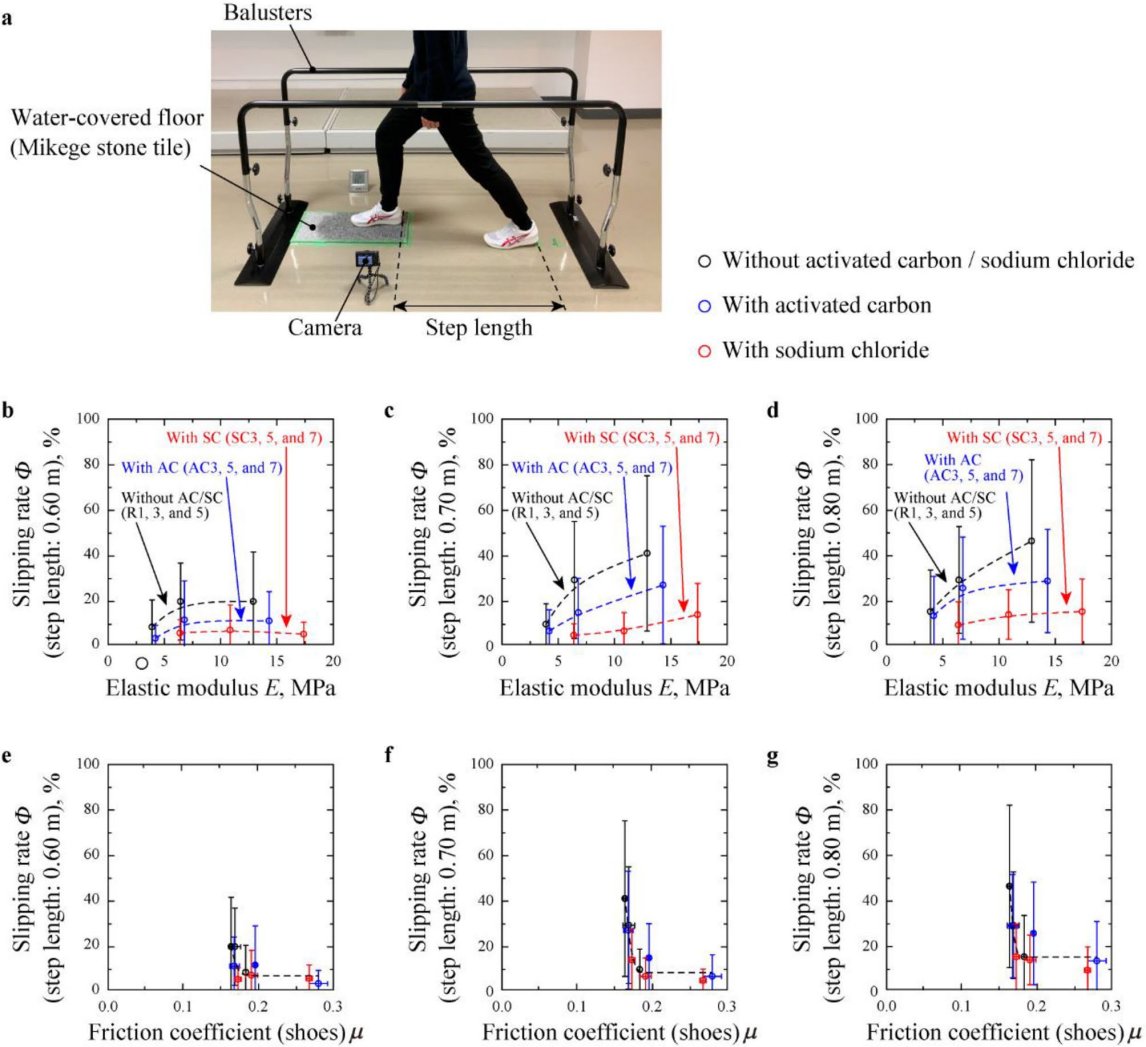
Overview and parameters obtained in slip-resistance test using shoes. (**a**) Experimental system under stepping motion. (**b**–**d**) Slipping rate (*Φ*) versus elastic modulus (E) for R1, R3, R5, AC3, AC5, AC7, SC3, SC5 and SC7. (**e**–**g**) *Φ* versus friction coefficient (*μ* using shoes with outer soles of R1, R3, R5, AC3, AC5, AC7, SC3, SC5 and SC7.

The slip rate of each outsole materials in stepping motion (Fig. 5a) was measured. Figure 5b–d reveal slipping rate *Φ* of R1, R3, R5, AC3, AC5, AC7, SC3, SC5 and SC7 outsole materials as a variation for *E* at the step length of 0.60, 0.70 and 0.80 m, respectively. It was observed that the *Φ* for all outsole materials increased with increase in *E*, and this dependency got more significant with an increase in the step length, especially for rubber without AC and SC. In addition, it was observed that *Φ* decreased with the addition of AC and SC. Figure 5e–g indicate the relationship between *Φ* and *μ* for footwear. The *Φ* values drastically decreased at *μ* < 0.19 and remained in steady values at *μ* ≥ 0.19 regardless of step length, and the convergence value of *Φ* at *μ* ≥ 0.19 increased with step length. These results indicate that the higher *μ* values provide the lower risk of slipping at *μ* < 0.19, and that the risk of slipping at *μ* ≥ 0.19 was not zero and increased with step length due to the uncertainty in human motion such as the stepping speed, the angle of heel contact, lean angle of body, and so on. Focusing on the *Φ*–*μ* curves at *μ* < 0.19, the decreasing rate of *Φ* decreased and got almost zero at *μ* = 0.19. Here, it is considered that the value of *Φ* was also depended on accidental changes in human motion, and that these accidental changes would be described by the Gauss distribution. Assuming that the relationship between *Φ* and *μ* for footwear is related to a Gaussian function, this relationship can be described by the mean value of friction coefficient *μ*_0_, the standard deviation of friction coefficient *σ*, and the convergence value of slipping rate *Φ*_0_, as shown in the following equation:

9 $$\Phi = \left( {1 - \Phi_{0} } \right)\frac{2}{\sqrt \uppi }\mathop \int \limits_{{\frac{{\mu - \mu_{0} }}{\sigma }}}^{\infty } e^{{ - t^{2} }} {\text{d}}t + \Phi_{0} = \left( {1 - \Phi_{0} } \right){\text{erfc}}\left( {\frac{{\mu - \mu_{0} }}{\sigma }} \right) + \Phi_{0} .$$

The values of *μ*_0_, *σ*, and *Φ*_0_ for each stepping length condition were determined by fitting the measured and estimated values of *Φ* based on a least squares method. The calculated *Φ*–*μ* curves are plotted as dash lines in Fig. 5e–g, and the calculated values of *μ*_0_, *σ*, and *Φ*_0_ are listed in Table 2. Figure 5e–g show that measured results were plotted on the fitting curves, and that all plots including error bar are plotted on these fitting curves. Here, *Φ* drastically decreased at *μ*_0_ + 3*σ* > *μ* ≥ *μ*_0_–3*σ* and got saturated at *μ* ≥ *μ*_0_ + 3*σ*. Since *μ*_0_ + 3*σ* = 0.181–0.189 as shown in Table 2, it is desirable to make *μ* > 0.189 to minimize the slipping risk in stepping motion. Figure 4e indicates that *μ* of footwear was > 0.189 for rubbers filled with AC and SC at *E* < 10 MPa. For the outsole containing the same master batch MB3 (R3, AC5, and SC5), the measured values of *μ* using footwear were 0.169, 0.196 and 0.191, respectively. Focusing on the case when the step length was 0.70 m (Fig. 5f), *Φ* was 28.6% for R3, and decreased to 14.1% and 5.9% with addition of AC and SC, respectively. Moreover, *Φ* for AC5 and SC5 further decreased to 5.9% and 4.1%, respectively, at *E* < 10 MPa. In summary, it is expected that the addition of AC or SC to the soft rubber (especially for *E* < 10 MPa) will be useful to reduce slipping risk in a practical use of footwear, at least in a stepping motion.

**Table 2:** Calculated parameters in *Φ*–*μ* curves.

Stepping length (m)	0.60	0.70	0.80
Mean value of friction coefficient *μ*_0_	0.152	0.161	0.162
Standard deviation of friction coefficient *σ*	0.0113	0.00945	0.00651
Convergence value of slipping rate *Φ*_0_	0.0622	0.0748	0.145
*μ*_0_ + 3*σ*	0.186	0.189	0.181

## Methods

### Material preparation

Rubbers were prepared in three steps: The compounds were mixed at 100–130 °C for 5.5 min (first mixing process) using a kneader (DS3–10MWB, Nihon Spindle Manufacturing Co., Ltd., Amagasaki, Japan) and continued to be mixed at low temperatures of 30–50 °C (second mixing process) using open roll (KD-M2-8, Kneader Machinery Co., Ltd., Taiwan, China) and finally pressed at 160 °C for 10 min (moulding process) using three types of moulds: sheet (215 mm × 130 mm × 2.0 mm), hemisphere (radius of curvature: 5.0 mm) and outer-sole. As shown in Table 3, five rubber compositions (labelled as MB1–5) were prepared in the first mixing process using isoprene rubber (Nipol IR2200, Zeon Corporation, Tokyo, Japan), silica (Nipsil VN3, Tosoh Silica Corporaion, Tokyo, Japan), oil (P200, Sineikako Co., Ltd., Kobe, Japan), Bis(triethoxysilylpropyl)tetrasulfide (Si69, Evonik Industries Japan, Osaka, Japan), Stearic acid (50S, New Japan Chemical Co., Ltd., Osaka, Japan) and zinc oxide (activated zinc oxide No.2, Honjo Chemical Corporation, Osaka, Japan). The content of silica was controlled to change *E*. In the second process, surfer (#200, Hosoi Chemical Co., Ltd., Oita, Japan), benzothiazolyl disulphide (Nocceler DM, Ouchi Shinko Chemical Industrial Co., Ltd., Tokyo, Japan), tetramethylthiuram monosulfide (Nocceler TS, Ouchi Shinko Chemical Industrial Co., Ltd., Tokyo, Japan), titanium oxide (A150, Sakai Chemical Industry Co., Ltd., Osaka, Japan), activated carbon AC (Shirosagi C SS, Osaka Gas Chemicals Co., Ltd., Osaka, Japan) and Sodium chloride SC (Nakuru UM-10, Naikai Salt Industries Co., Ltd.,, Okayama, Japan) were added to the composites (MB1–5) obtained in the first mixing process. Here, according to the AC maker, this AC was made from wood, the peak of distribution of holes on AC surface was about 2 nm, the median size of AC was about 50 μm, the specific surface area was about 900 m^2^/g, and the AC surface was not chemically treated. As to the SC, the purity of SC was 99.59%, and the median size of SC was 11.1 μm. Rubbers without AC or SC (labelled as R1–5) were prepared using MB1–5, respectively. While the rubbers filled with AC (labelled as AC3, AC4, AC6 and AC7) and the rubbers filled with SC (labelled as SC3, SC4, SC6 and SC7) were made from MB1, MB2, MB4 and MB4, respectively, AC1, AC2, AC5, AC8, SC1, SC2, SC5, SC8 and SC9 were prepared based on MB3 with different content of AC or SC as shown in Table 1. Titanium oxide was added to each rubber to ensure the high intensity of reflected light in friction test using hemisphere specimens. Even the change in physical properties (except density) by adding titanium oxide was negligible, titanium oxide was not added for outsole preparation to compare friction behaviour of outsoles without titanium oxide, because titanium oxide is not always added to outsole of shoes for sale. To control pore size for rubber filled with SC, we used sodium chloride grade with controlled size (≈ 10 μm). Table 1 also shows the physical properties of each rubber. Tensile strength and tear strength were measured based on ISO 37:2017 and ISO34-1:2004, respectively, using dumbbell-like specimens and angle test piece with ​a nick of specified depth, respectively, by setting head speed at 500 mm/min. *E* was defined as the average elastic modulus when strain was < 5.0% in the tensile strength measurement. For hemisphere specimens, radius of curvature and arithmetical mean height *S*_a_ were measured using a One-Shot 3D measuring macroscope (VR3000, Keyence Corporation, Osaka, Japan). *S*_a_ was quantified based on the 1.000 μm square within bottom top of hemisphere specimens where plane correction was applied in the accompanying software (VR-H1A, Keyence Corporation, Japan). It was confirmed that the radius of curvature and *S*_a_ were within 5.21–5.29 mm and 1.21–1.52 μm, respectively, among all hemisphere specimens. Surface free energy of each rubber was quantified by contact angle with 1.0-μL ion-exchanged water and diiodomethane (Wako 1st grade, FUJIFILM Wako Pure Chemical Corporation, Osaka, Japan) using a contact angle meter (DMs-401, Kyowa Interface Science Co., Ltd., Saitama, Japan)^33^. The dispersion and polar terms of free energy were within 0.0–0.1 mJ/m^2^ and 24.1–26.0 mJ/m^2^, respectively. According to the geometrical properties and surface free energy, the difference in these parameters were so minute that real contact formation and friction behaviour could not be affected by these parameters. Figure 1a indicates overall images of hemispheres specimens (R3, AC5 and SC5) and their scanning electron microscope (SEM) images observed by a SEM (JSM-6390A, JEOL Ltd., Tokyo, Japan). Here, for hemisphere specimens and outsole rubber materials filled with SC, the sodium chloride particles on rubber surface was removed by washing in water and drying at room temperature before surface observation and friction tests. SEM images in Fig. 1a explain that air pockets were formed by adding AC and SC, and that the air pocket size was about 0.1 μm and 5 μm, respectively. Using outsoles of all rubbers, footwear (TMM800, size: 27.0 cm, ASICS Corporation, Kobe, Japan) were prepared in a shoe maker (Sanin ASICS Industry Corporation, Sakaiminato, Japan) as shown in Fig. 4a–c.

**Table 3:** Rubber compositions in the first mixing process (unit: phr).

		MB1	MB2	MB3	MB4	MB5
Polymer	Isoprene rubber	100	100	100	100	100
Reinforcing filler	Silica	44	51	60	69	80
Plasticizing agent	Oil	20	20	20	20	20
Silane coupling agent	Bis(triethoxysilylpropyl) tetrasulfide	4.4	5.1	6.0	6.9	8.0
Processing aid	Stearic acid	2.0	2.0	2.0	2.0	2.0
	Zinc oxide	5.0	5.0	5.0	5.0	5.0
Others (antioxidant and vulcanization accelerator)		3.5	3.5	3.5	3.5	3.5

### Friction test using hemisphere specimens

A friction test was conducted using all hemisphere specimens (R1–5, AC1–8 and SC1–9) and contact condition was observed during the friction test. Figure 1b shows a schematic view of experimental system. Each hemisphere specimen perpendicularly approached to a water-covered glass surface (084.4L100-45DEG-6P-4SH3.5, SIGMAKOKI Co., Ltd., Saitama, Japan) at 1.00 mm/s using an electric cylinder (EASM4NXD010AZMC, Oriental Motor Co., Ltd., Japan) and within 0.01 s after completion of contacting process. The glass was slid horizontally and linearly at 1.00 mm/s using another electric cylinder (EACM4D30AZAC, Oriental Motor Co., Ltd., Japan). Normal force was set at 0.196 N using the dead weight as shown in Fig. 1b, and friction force was measured using a load cell (TL201Ts, Trinity-Lab Inc., Tokyo, Japan) at 1 kHz. In the contact-condition observation, distributions of water, air and real contacts were measured based on intensity in a total reflection method and a light interferometry^11^, and distribution of clearance between rubber and glass was quantified based on the intensity in the total reflection method^34^. As shown in Fig. 1b, in the total reflection method, red light from a light-emitting diode (LED, HLV2-22RD-3 W, CCS Inc., Kyoto, Japan) was penetrated into the glass as totally reflected in the glass (reflect angle was set at 65°) using a light guide (LE-OPT-24, OPTEC FA Co., Ltd., Kyoto, Japan) and a mirror (RPB3-20-550, SIGMAKOKI Co., Ltd., Saitama, Japan). In the light interferometry, blue light form LED (HLV2-22BL-3W, CCS Inc., Kyoto, Japan) was perpendicularly inserted in the interface between hemisphere specimen and glass plate through a telecentric lens (TV-2F-110, OPTART Co., Ltd., Tokyo, Japan). The reflected light was observed by a charge-coupled device camera (AT-030MCL, JAI Ltd., Yokohama, Japan) at 12 bit and 100 fps in both total reflection method and light interferometry. The pixel size corresponded to 3.6 μm × 3.6 μm. In this study, friction and observation test for each condition was conducted five times. The friction force and contact condition were measured at *d* = 0.00–10.00 mm, and mean values and standard deviation of each parameter were obtained from the results measured at *d* = 5.00–10.00 mm. Atmosphere temperature and relative humidity were set at 21.8 °C and 68%, respectively.

### Friction test using footwear with a slip meter

The friction test was conducted with a slip meter (SATRA TM144, SATRA Technology Centre Ltd., Kettering, UK) based on ISO 13287 using right footwear with all rubbers, as shown in Fig. 4d. As the toe of footwear was not in contact with a water-covered smooth Mikage stone tile (G603 White, Sakae shokai Co., Ltd., Tajimi, Japan), the angle between outsole and floor was set at 7°. The surface roughness of the floor was measured by a roughness meter (SV-3000S4, Mitutoyo Corporation, Kawasaki, Japan) and *S*_a_ = 0.070 μm. Normal force, sliding velocity and sliding distance *d* were set at 500 N, 300 mm/s and 200 mm respectively. Based on ISO 13287, friction test was conducted 10 times for each outsole materials, and mean value and standard deviation of friction coefficient *μ* was calculated from the results in 6th–10th trials at *d* = 100–200 mm. Atmosphere temperature and relative humidity were 22.6 °C and 37%, respectively.

### Slip-resistance test using shoes in a stepping motion

The slip resistance in stepping motion was measured as shown in Fig. 5a using right footwear with outsole of R1, R3, R5, AC3, AC5, AC7, SC3, SC5 and SC7. Because *μ* of shoes was especially sensitive to *E* and addition of AC or SC in comparison with the content of these addition as shown in Fig. 4e,f, the footwear with R1, R3, R5, AC3, AC5, AC7, SC3, SC5 and SC7 were selected to conduct this test. The water-covered smooth Mikage tile was used as the floor, as well as in the friction test using shoes. Eleven healthy young male adults (age: 22–36 years, height: 1.65–1.91 m, body mass: 52–76 kg, dominant foot: right or left) participated in this trial. All methods/experiments were performed in accordance with relevant guidelines and regulations. The participants were informed of the protocol, and informed consent was obtained from each participant before the experiment. The protocol was approved by the Institutional Review Board of Tohoku University. The participants were asked to wear each footwear and stand still. Then, they were asked to take a single forward step on the floor with a step length of 0.6, 0.7 and 0.8 m. Here, subjects were instructed to make heel contact with the floor in the stepping motion. While the stepping motions were conducted continuously 20 times for each outsole-step length condition, the order of the trial condition was randomized. The contact between shoe and floor in the stepping motion was observed by a digital camera (PowerShot SX700 HS, Sony, Tokyo, Japan) at 60 fps. In this chapter, whether a slip occurred or not was defined as whether the slipping distance was more than 0.03 m or not, based on the previous study^35^. To easily judge the slipping distance by moving during the stepping motion, parallel lines orthogonal to the stepping direction were printed par 1.0 cm on the floor. Mean value and standard division of slip rate for each condition (9 types of outer-sole and 3 grades of step length) were quantified based on the results of 220 steps (= 11 subjects × 20 steps). To avoid a serious slip-and-fall accident during this test, balusters (P-2, Mutsumi Medical Co., Ltd., Osaka, Japan) were set on the both side of subjects; however, no subjects touched these balusters before the heel contacts in stepping motion. The temperature and relative humidity were 17.0 °C and 33%, respectively.
